# Prenatal maternal stress, breastfeeding and offspring ADHD symptoms

**DOI:** 10.1007/s00787-024-02451-5

**Published:** 2024-04-30

**Authors:** Jandeh Jallow, Tuula Hurtig, Martta Kerkelä, Jouko Miettunen, Anu-Helmi Halt

**Affiliations:** 1 Research Unit of Clinical Medicine, Psychiatry, University of Oulu, Sairaalanrinne 2 A 32, Oulu, 90220 Finland; 2 Medical Research Centre Oulu, Department of Psychiatry, Oulu University Hospital and University of Oulu, Oulu University Hospital, Oulu, Finland; 3 Clinic of Child Psychiatry, Oulu University Hospital, Oulu, Finland; 4 Research Unit of Population Health, University of Oulu, Oulu, Finland; 5 Department of Psychiatry, Oulu University Hospital, Oulu, Finland

**Keywords:** ADHD, Prenatal maternal stress, Unwanted pregnancy, Breastfeeding

## Abstract

There is increasing evidence to suggest that environmental factors are associated with ADHD, but results regarding prenatal maternal stress, unwanted pregnancy, breastfeeding, and ADHD in children are controversial and few prospective studies have been conducted. Using prospectively collected data from the Northern Finland Birth Cohort 1986 (*n* = 7,910) we studied potential risk factors for ADHD symptoms at 8 and 16 years of age, including prenatal maternal stress and unwanted pregnancy, and protective factors including the duration of breastfeeding. Prenatal stress was associated with an increased risk of ADHD symptoms at the age of 16 (OR = 1.95, 95% CI: 1.34–2.80) and an unwanted pregnancy correlated with hyperactivity symptoms in the offspring at the age of 8 (OR = 2.08, 95% CI: 1.55–2.77). We did not find an association between prenatal maternal stress and hyperactivity symptoms in the offspring at the age of 8 (OR = 0.87, 95% CI: 0.69–1.08) or with unwanted pregnancy and ADHD symptoms at the age of 16 (OR = 1.13, 95% CI: 0.57–2.02). In relation to breastfeeding, over three months of exclusive breastfeeding was associated with lower hyperactivity symptoms in the 8-year follow-up (OR = 0.65, 95% CI: 0.46–0.92) and there was evidence of same kind of relationship concerning non-exclusive breastfeeding, but the association was not statistically significant (OR = 0.76, 95% CI: 0.54–1.06). In 16-year follow-up, under six months of non-exclusive breastfeeding showed an association with ADHD symptoms (OR = 0.68, 95% CI: 0.48–0.95) while exclusive breastfeeding did not (OR = 1.00, 95% CI: 0.66–1.55). In conclusion, our findings suggest that prenatal maternal stress increases the risk of more severe forms of ADHD symptoms in the offspring and breastfeeding can protect against such symptoms at the ages of 8 and 16.

**Supplementary Information:**

The online version contains supplementary material available at 10.1007/s00787-024-02451-5.

## Introduction

Attention-deficit/hyperactivity disorder (ADHD), one of the most common neurobehavioural disorders, is a multifactorial condition defined by age-inappropriate symptoms of attention problems, hyperactivity and impulsivity leading to functional impairments in at least two operational environments, including education and social relationships. ADHD has an estimated childhood prevalence of 4–7% [1], with increasing evidence pointing to its continuation into adulthood for between 15% and 65% of individuals with an estimated prevalence of 2.5% [2]. In Finland, around the time when the ADHD symptom data for this study was collected, prevalence for stimulant use for ADHD was 0.6% [3].

While it is well accepted that the disorder is highly heritable (> 70%) [4], not all the risk is genetic. It is estimated that between 10 and 40% of the variance associated with ADHD is likely to be accounted for by environmental factors [5]. In view of this, there is a growing literature examining the association between prenatal maternal stress and the risk of ADHD in the offspring. Stress in this context includes negative life events, fatigue, anxiety and depressive symptoms and the prevalence of such stress in pregnancy has been determined to be 31% [6]. Previous studies have been inconsistent, however, as to whether the offspring of mothers who experience high levels of stress during pregnancy are more likely to have ADHD or not. While many studies, including meta-analysis made in 2019, have shown that prenatal maternal stress increases the risk of the offspring developing ADHD [7, 8], not all findings have been in line with this [9, 10]. Furthermore, there are limitations in the literature on prenatal maternal stress and ADHD. To this day, there are only few prospective studies made while majority of the studies reporting adjusted results are case–control studies, which may be subject to recall bias. In addition, most of the studies do not adjust for key potential confounders such as gender, parents’ psychiatric disorders, maternal age, and socio-economic status. In this study, we use fatigue to determine prenatal maternal stress. Evidence suggests a clear bidirectional association between fatigue and stress [11].

Unwanted pregnancy, a negative attitude towards the pregnancy, is a pregnancy-specific stressor which is large factor affecting mental health of the pregnant women. Unwanted pregnancy has been connected with offspring poorer neurodevelopment [12] and children born after unwanted pregnancy are associated with psychiatric disorders, including schizophrenia-spectrum and affective disorders [13]. There are no previous prospective studies concerning unwanted pregnancy and offspring ADHD.

Breastfeeding is one of the most widely studied and important postnatal factors influencing not only infant health but also early cortical development, and thus probably self-regulation and neurodevelopmental conditions. A lack of breastfeeding has been linked with offspring mortality caused by infectious diseases, child obesity, both type 1 and type 2 diabetes mellitus, leukaemia, and sudden infant death syndrome [14]. As for the importance of breastfeeding, the Finnish Institute of Health and Welfare recommends exclusive breastfeeding for the first 4 to 6 months, with continued breastfeeding along with the introduction of appropriate complementary foods up to one year of age [15].

The duration of breastfeeding can be expected to be particularly important among the offspring’s early experiences as far as ADHD is concerned. Although no actual association has been established, some studies have shown that ADHD is more common in children with lower rates of breastfeeding [16], although the targeting of either exclusive or non-exclusive breastfeeding might account for the differing results [17, 18]. Also, it is not clear how a possible association could be explained: do the breastfeeding and the breast milk influence the child´s development or are ADHD children just harder to breastfeed. In any case, several recent studies have found no association between maternal breastfeeding and offspring ADHD [19, 20].

Early predictors of ADHD are critically needed, as they could inform aetiological theory and perhaps help identify new prevention targets. Results regarding prenatal maternal stress, unwanted pregnancy, breastfeeding and ADHD in children are controversial, and few prospective studies have been conducted. Given the above-mentioned gaps in the literature, the aims of this work were to see whether there are any associations of risk factors, including prenatal maternal stress and unwanted pregnancy, and protective factors including the duration of breastfeeding with ADHD symptoms in childhood and adolescence. For this purpose, we used a large prospective population-based sample from the Northern Finland Birth Cohort 1986 (NFBC1986).

## Methods

### Population

**Fig. 1:**
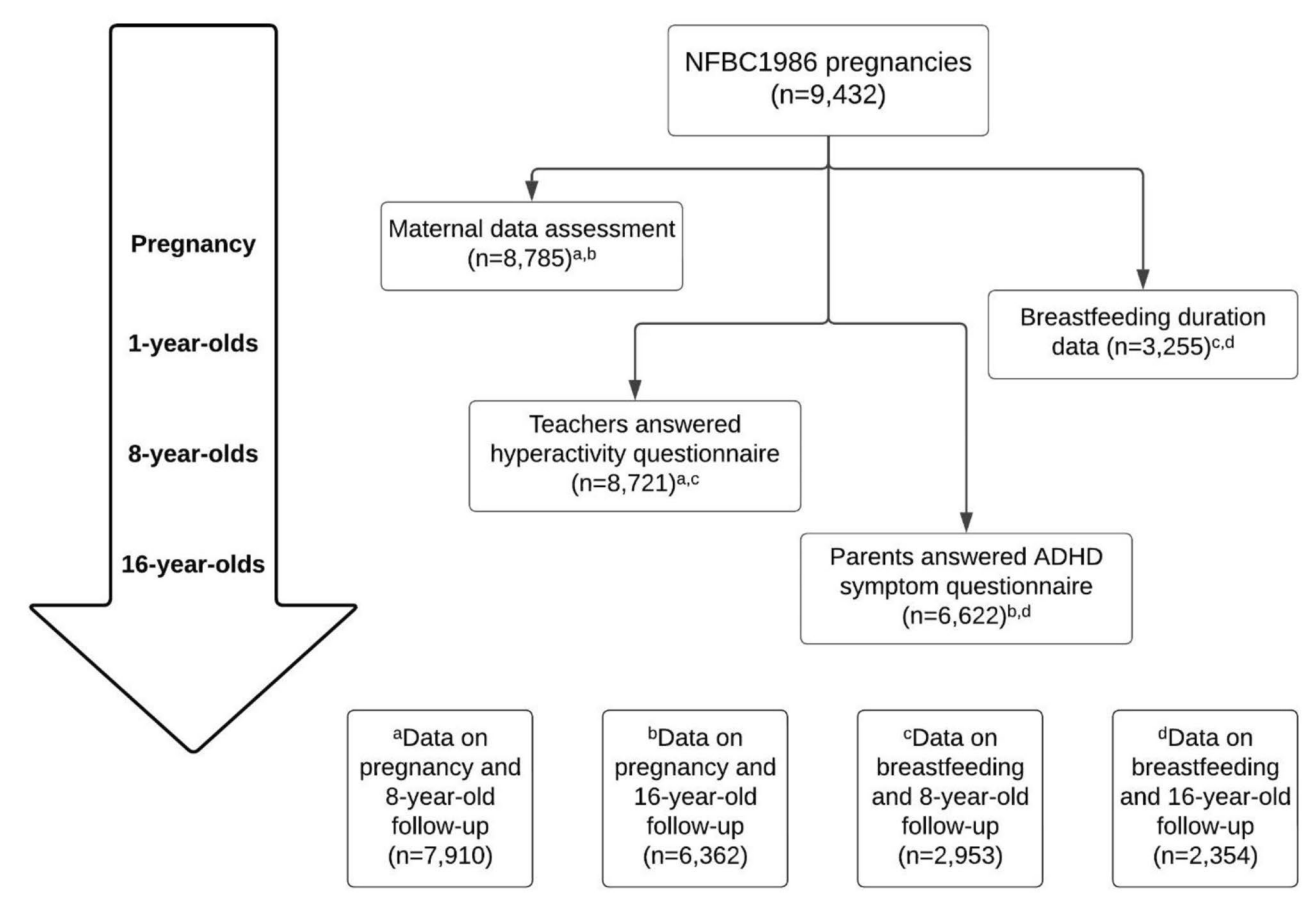
Flowchart of the investigation Maternal data assessment: a questionnaire including information on maternal fatigue during pregnancy and the desirability of the pregnancy

The population consisted of the Northern Finland Birth Cohort 1986 (NFBC1986), a prospective mother-child cohort that includes 99% of all births that took place in the area concerned in the interval July 1st, 1985 - June 30th, 1986 (*n* = 9,479, live born 9,432) [21, 22]. 8785 mothers completed a questionnaire including information on fatigue (i.e. stress) during pregnancy and the desirability of the pregnancy. Alongside this assessment, questionnaires on ADHD symptoms in the offspring were sent to their teachers at age 8 (*n* = 7,910) and their parents at age 16 (*n* = 6,362). Breastfeeding data were available for 3,255 cases, of which ADHD symptom questionnaires at ages of 8 and 16 years were available for 2,953 and 2,354, respectively (Fig. 1).

### Instruments and measures

#### ADHD symptoms

Symptoms of ADHD in childhood were evaluated by the child’s main teacher at eight years of age (*n* = 7,910). The teachers filled in a questionnaire that included the official Finnish translation of the Rutter scale B2 with 26 items (three of these concerning hyperactivity). The total score on the hyperactivity items range from zero to six, and the children were categorized as hyperactive if they scored three points or more [23]. The Rutter B2 scale has been validated as a screening instrument for ADHD symptoms among a nationwide population sample of 8-year-old Finnish children [24].

When the participants reached the age of sixteen their parents were asked to complete a questionnaire that included the Strengths and Weaknesses of ADHD Symptoms and Normal Behaviour (SWAN) Scale to detect ADHD symptoms [25] (*n* = 6,362). This is based on the 18 ADHD symptoms described in DSM-IV-TR [26] and evaluates these symptoms on a seven-point scale, so that the 95th percentile of the score distribution on this scale can be used as a cut-off point to define adolescents with ADHD symptoms (inattentive, hyperactive-impulsive or both) [27]. SWAN scores distinguish ADHD participants with high sensitivity and specificity [28]. We divided the adolescents into three mutually exclusive groups based on the SWAN symptoms: inattentive, hyperactive-impulsive, and combined (adolescents defined as both inattentive and hyperactive-impulsive cases). In the analyses of breastfeeding, we compiled a SWAN symptom category consisting of adolescents with inattentive and/or hyperactive-impulsive symptoms.

#### Pregnancy and breastfeeding

The mothers had been asked before the 24th week of the pregnancy to fill in a questionnaire on the desirability of the pregnancy. We divided the answers into two categories: “planned pregnancy” and “unplanned or planned later”. At the last visit to antenatal clinics or within a week of giving birth, the mothers answered a question concerning how fatigued they had felt during the pregnancy (better than before, ordinary, fatigued), we then further classified the answers into two groups (0 = ordinary or better than before, 1 = fatigued).

When the children reached the age of one year their health clinic cards had been collected and their mothers were asked to fill in a questionnaire on the duration of breastfeeding. This information, with the clinic card data prioritized, was used to determine the duration of exclusive and non-exclusive breastfeeding. We then divided the exclusive breastfeeding cases into two categories: duration under three months and three months or more, and the non-exclusive breastfeeding similarly into two groups: duration under six months and six months or more.

#### Sociodemographic factors

The parents of the NFBC1986 children had also filled in a questionnaire during pregnancy concerning relevant sociodemographic factors: their marital (family structure) and social status, education, work, health and living habits. We then divided the educational status replies into two categories: parents without any high school (HS) education (only primary education) and those with a high school, college, or university education. Similarly, we categorized maternal ages into three groups: under 20 years, from 20 to 35 years and over 35 years. Parents’ marital status was divided into two categories: two parents (married or living together) and one parent (unmarried, widow, divorced). We also included maternal and parental psychiatric disorders in our material provided they had been diagnosed before the offspring reached the age of 8 in the 8-year follow-up or age of 16 in the 16-year follow-up.

### Statistical methods

Associations between unwanted pregnancy, prenatal maternal fatigue, duration of breastfeeding and offspring ADHD symptoms were investigated using logistic regression. Odds ratios (OR) are reported with 95% Confidence intervals (CI). In the analyses for prenatal maternal fatigue and unwanted pregnancy, we categorize the ADHD symptoms in four categories: hyperactivity symptoms at the age of 8 and hyperactive-impulsive, inattentive, and combined ADHD symptoms at the age of 16. In the analyses of breastfeeding, we used Rutter B2 scale for hyperactivity symptoms at the age of 8 and SWAN symptom category consisting of 16-year-olds with inattentive and/or hyperactive-impulsive symptoms. In further analyses, associations between unwanted pregnancy, prenatal maternal fatigue, and continuance of the ADHD symptoms continuance we categorized ADHD symptoms into three categories: positive ADHD symptoms (hyperactivity symptoms) only at the age of 8, positive ADHD symptoms only at the age of 16 (inattentive and/or hyperactive-impulsive symptoms) and positive ADHD symptoms at both time points.

We assessed crude models as well as adjusted models with information of the gender of the offspring, mother´s high school education, mother´s age at the time of the labour and maternal psychiatric disorders. Additionally, we used a model that adjusted desirability of the pregnancy and maternal prenatal fatigue. In adjusted supplementary tables we also included parents’ marital status. The frequencies of the sociodemographic factors in the offspring grouped by ADHD symptoms are reported and comparisons are made using Pearson’s chi-square test (χ2).

## Results

The results for sociodemographic factors at the 8-year follow-up are shown in Table 1. Comparing to the controls, the hyperactive cases were more often boys (*p* < 0.001) and had only one parent (*p* < 0.001) and their mothers more often gave birth under the age of 20 (*p* < 0.001) and had no high school education (*p* = 0.001). A history of unwanted pregnancy was significantly higher among the children with hyperactivity than in the control subjects (*p* < 0.001). The groups did not differ in terms of maternal fatigue (*p* = 0.228) or parental psychopathology. Furthermore, ADHD hyperactivity symptoms at the age of 8 were associated with all the ADHD symptom domains at the age of 16, the hyperactive individuals having all the ADHD symptoms more often than the controls (not shown in the table).

**Table 1:** Sociodemographic factors of the 8-year follow-up

	No hyperactive symptoms	Hyperactive symptoms	χ2
n	7859	630	*p*-value
Gender			< 0.001
Male	3833 (48.8)	510 (81.0)	
Female	4026 (51.2)	120 (19.0)	
Unwanted pregnancy	513 (7.6)	81 (15.1)	< 0.001
*Missing*	*1081*	*93*	
Fatigue	1417 (22.3)	105 (19.9)	0.228
*Missing*	*1503*	*103*	
Only primary education	1761 (25.7)	176 (32.6)	0.001
*Missing*	*997*	*90*	
Mother’s age during labour			
<20	309 (3.9)	52 (8.3)	< 0.001
20–35	6735 (85.7)	510 (81.0)	
>35	815 (10.4)	68 (10.8)	
Marital status			
Two parents	7469 (95.3)	574 (91.4)	< 0.001
One parent	368 (4.7)	54 (8.6)	
*Missing*	*22*	*2*	
Maternal psychiatric disorder	452 (5.8)	38 (6.0)	0.840
Paternal psychiatric disorder	447 (5.7)	43 (6.8)	0.276

The sociodemographic factors observed among the adolescents with ADHD symptoms and their controls in the 16-year follow-up data are shown in Table 2. Here again, the males showed more ADHD features than the females in all the symptom domains, except that the groups did not differ in terms of the desirability of the pregnancy, parents’ marital status or paternal psychiatric disorders. The mothers of the adolescents with combined ADHD symptoms (*p* = 0.002) presented with more psychiatric disorders than those in the control group. Moreover, the mothers of the control cases were better educated than those of the combined ADHD symptom cases, as they more often had a high school education or higher degree. It is also clear from Table 2 that the mothers of the children who showed combined ADHD symptoms had felt fatigue more often during pregnancy than those of other groups. In addition, the children whose mothers had given birth under the age of 20 more often had inattentive symptoms comparing to other ADHD symptom groups and the control group. Further, observations on associations between maternal age, fatigue, and unwanted pregnancies were that mothers aged over 35 years were more often fatigued than under 35-year-olds and those aged under 20 years had more unwanted pregnancies than older mothers (Supplementary Table 1).

**Table 2:** Sociodemographic factors in the 16-year-follow up

	No ADHD symptoms	Combined ADHD symptoms	χ2	Inattentive ADHD symptoms		χ2		Hyperactive-impulsive ADHD symptoms		χ2	
	(*n* = 6194)	(*n* = 150)	*p*-value	(*n* = 130)		*p*-value		(*n* = 299)		*p*-value	
	n (%)	n (%)		n (%)	n (%)						
Gender											
Male	3026 (48.9)	104 (69.3)	< 0.001	87 (66.9)		< 0.001		174 (58.2)		0.002	
Female	3168 (51.1)	46 (30.7)		43 (33.1)				125 (41.8)			
Unwanted pregnancy	393 (7.3)	11 (8.1)	0.822	11 (10.6)		0.273		17 (6.6)		0.765	
*Missing*	*780*	*15*		*26*				*40*			
Fatigue	1110 (21.8)	45 (35.2)	< 0.001	23 (23.2)		0.818		53 (21.7)		1.000	
*Missing*	*1092*	*22*		*31*				*55*			
Only primary education	1280 (23.4)	44 (31.4)	0.034	33 (31.7)		0.070		70 (26.6)		0.254	
*Missing*	*715*	*37*		*45*				*36*			
Mother’s age during labour											
<20	207 (3.3)	10 (6.7)	0.082	11 (8.5)		0.006		11 (3.7)		0.585	
20–35	5291 (85.4)	125 (83.3)		107 (82.3)				260 (87.0)			
>35	696 (11.2)	15 (10.0)		12 (9.2)				28 (9.4)			
Marital status											
Two parents	5925 (95.8)	140 (93.3)	0.200	123 (95.3)		0.970		284 (87.0)		0.992	
One parent	259 (4.2)	10 (6.7)		6 (4.7)				13 (4.4)			
*Missing*	*10*	*0*		*1*				2			
Maternal psychiatric disorder	292 (4.7)	16 (10.7)	0.002	11 (8.5)		0.076		18 (6.0)		0.371	
Paternal psychiatric disorder	311 (5.0.)	10 (6.7)	0.471	11 (8.5)		0.118		23 (7.7)		0.056	

The maternal and pregnancy-related risk factors for ADHD are shown in Table 3. Here the crude model shows an association between prenatal maternal fatigue and combined ADHD symptoms at the age of 16, an association that remained significant after adjustment (OR = 1.88, 95% CI: 1.26–2.77). Similarly, an unwanted pregnancy was related to hyperactivity symptoms in the offspring at age 8 years in both models (OR = 2.08, 95% CI: 1.55–2.77). Furthermore, children whose mother had a lower level of education showed more hyperactivity symptoms at the age of 8 (OR = 1.27, 95% CI: 1.02–1.57) and combined ADHD symptoms at the age of 16 (OR = 1.56 95% CI: 1.04–2.32).

**Table 3:** Maternal and prenatal risk factors for ADHD symptoms at the ages of 8 and 16 in the crude and adjusted models

	Combined ADHD symptoms at age 16	Inattentive ADHD symptoms at age 16	Hyperactive-impulsive ADHD symptoms at age 16	Hyperactive symptoms at age 8
Crude models	OR (95% CI)	OR (95% CI)	OR (95% CI)	OR (95% CI)
Unwanted pregnancy	1.13 (0.57–2.02)	1.51 (0.76–2.72)	0.9 (0.52–1.44)	2.17 (1.67–2.78)**
Fatigue	1.95 (1.34–2.80)**	1.09 (0.67–1.71)	1.00 (0.72–1.35)	0.87 (0.69–1.08)
Adjusted models				
Unwanted pregnancy	1.01 (0.47–1.94)	1.43 (0.65–2.80)	0.74 (0.39–1.30)	2.08 (1.55–2.77)**
Fatigue	1.88 (1.26–2.77)*	1.34 (0.97–1.84)	1.02 (0.73–1.41)	0.78 (0.61-1.00)
Gender (female ref.)	2.50 (1.69–3.77)**	2.21 (1.41–3.54)**	1.50 (1.14–1.98)*	4.21 (3.36–5.33)**
Only primary education	1.56 (1.04–2.32)*	1.46 (0.90–2.31)	1.23 (0.89–1.67)	1.27 (1.02–1.57)*
Mother´s age during labour				
<20	1.76 (0.72–3.71)	2.00 (0.75–4.49)	1.50 (0.72–2.81)	1.88 (1.26–2.72)*
>35	0.50 (0.21-1.00)	1.13 (0.54–2.12)	0.85 (0.52–1.33)	1.10 (0.78–1.51)
Two parents	0.56 (0.27–1.34)	2.42 (0.71–15.32)	1.19 (0.57–2.89)	0.80 (0.53–1.23)
Maternal psychiatric disorders	1.81 (0.79–3.59)	2.74 (1.19–5.48)*	0.89 (0.37–1.79)	0.97 (0.62–1.45)

* *p*-value < 0.05, **p-value < 0.001

Adjusted for the desirability of the pregnancy, maternal prenatal fatigue, gender of the offspring, mother´s education level, mother´s age at the time of labour, and maternal psychiatric disorders. OR = odds ratio, CI = confidence interval

Of the 3,255 mothers who exclusively breastfed, 578 (21.8%) breastfed for less than three months and 2072 (78,2%) breastfed over three months. For non-exclusive breastfeeding the values for under and over six months were 970 (43.6%) and 1257 (56.4%), respectively. Over three months of exclusive breastfeeding was associated with lower pronounced hyperactivity symptoms in the 8-year follow-up, even after adjustment (OR = 0.58, 95% CI: 0.40–0.85), but there was no significant association in the case of non-exclusive breastfeeding (OR = 0.76, 95% CI: 0.54–1.06). In addition, less than six months of non-exclusive breastfeeding was associated with a small but statistically significant increase in ADHD symptoms in the 16-year follow-up (OR = 0.65, 95% CI: 0.44–0.95). The relevant crude and adjusted breastfeeding models are shown in Table 4.

**Table 4:** Associations of exclusive and non-exclusive breastfeeding with ADHD symptoms at the ages of 8 and 16 years

Exclusive breastfeeding	Any ADHD symptoms at age 16	Hyperactive symptoms at age 8
Crude models	OR (95% CI.)	OR (95% CI.)
Breastfeeding ≥ 3 months	1.00 (0.66–1.55)	0.65 (0.46–0.92)*
Adjusted models		
Breastfeeding ≥ 3 months	1.17 (0.73–1.94)	0.58 (0.40–0.85)*
Gender (female ref.)	1.72 (1.19–2.52)*	3.77 (2.53–5.76)**
Only primary education	1.28 (0.82–1.95)	0.83 (0.53–1.25)
Non-exclusive breastfeeding		
Crude models	OR (95% CI.)	OR (95% CI.)
Breastfeeding ≥ 6 months	0.68 (0.48–0.95)*	0.76 (0.54–1.06)
Adjusted models		
Breastfeeding ≥ 6 months	0.65 (0.44–0.95)*	0.79 (0.54–1.16)
Gender (female ref.)	1.67 (1.14–2.47)*	3.82 (2.49–6.08)**
Only primary education	0.94 (0.58–1.47)	1.19 (0.77–1.81)

* *p*-value < 0.05 **p -value < 0.001

Adjusted for gender of the offspring, mother´s education level, and mother’s age at the time of labour. OR = odds ratio, CI = confidence interval

We did further analyses on a comparison of the difference between the outcomes of ADHD symptoms in both follow-ups and sociodemographic factors, prenatal maternal fatigue, unwanted pregnancy, and duration of breastfeeding. The results showed that prenatal maternal fatigue and female gender are risk factors for developing ADHD symptoms later in life and showing them only at the age of 16 compared to the individuals showing ADHD symptoms in both follow-ups and only at the age of 8 (Supplementary Table 2). Furthermore, we found an association between non-exclusive breastfeeding and prenatal maternal fatigue. Pregnant women who experienced fatigue were more likely to breastfeed non-exclusively shorter duration compared to the ones without fatigue. When seeking the combined effect of prenatal maternal fatigue, unwanted pregnancy and breastfeeding on ADHD symptoms, non-exclusive breastfeeding was no longer associated with ADHD symptoms at the age of 16. These results are shown in Supplementary Table 3.

## Discussion

To our knowledge, this large cohort study of risk factors for ADHD symptoms occurring at ages of 8 and 16 years is one of the first prospective population-based investigations into the association between prenatal stress and the risk of ADHD symptoms in childhood and adolescence. The overall findings showed an association between prenatal fatigue and an increased risk of combined ADHD symptoms at the age of 16, although fatigue was not associated with any other ADHD symptom at either time point. These results indicate that prenatal maternal stress is related to more severe forms of ADHD symptoms and may contribute to the development of symptoms that fulfil the diagnostic criteria for ADHD. This association is in line with previous findings of an association between prenatal maternal stress and ADHD [7], but it should be noted that this is the first cohort study taking confounding factors into account to find an association of ADHD with self-perceived maternal fatigue during pregnancy, while previous studies have found a significant association only in male offsprings or following maternal stress attributable to bereavement in the form of the unexpected death of a family member [7].

Breastfeeding is one of the most vital factors for the health of the offspring during the first year of life. The present findings regarding breastfeeding were inconclusive, however, in that under three months of exclusive breastfeeding was associated with higher hyperactivity symptoms in the 8-year follow-up but the same statistically significant relationship was not found where non-exclusive breastfeeding was concerned, while contrary to this, under six months of non-exclusive breastfeeding showed a correlation with ADHD symptoms in the 16-year follow-up whereas exclusive breastfeeding did not. The present finding of a shorter period of breastfeeding is in line with a few earlier observations that the distinction between exclusive and non-exclusive breastfeeding accounted for differences in the results [17, 18]. In addition, a previous meta-analysis has shown that the incidence of non-exclusive breastfeeding of 3–6 months’ duration did not bring about any significant difference in children with and without ADHD symptoms [16]. When studying the combined effect of prenatal maternal fatigue, unwanted pregnancy and breastfeeding on ADHD symptoms, non-exclusive breastfeeding was no longer associated with ADHD symptoms at the age of 16. This could be because the association between non-exclusive breastfeeding and ADHD symptoms at the age of 16 was through prenatal maternal fatigue while fatigued mothers non-exclusively breastfed shorter times than the controls.

Even so, most of the earlier literature suggests a clear relationship between ADHD and a lower breastfeeding rate. If breastfeeding is associated causally with ADHD, its effect is likely to be part of an interplay with genotype and the environment. Several studies of ADHD have suggested that genetic and environmental risk factors may act together, giving rise to the theory that some genes may influence the development of ADHD by affecting the individual’s sensitivity to environmental adversity, and that the long-term clinical course of ADHD is influenced by prenatal, biological and psychosocial environmental risk factors [29, 30]. In the light of these opinions, our results indicate that the causal relationship between a short duration of breastfeeding and ADHD is unclear and can be explained by factors that are related to both the child and the mother. The mothers in the ADHD symptom groups more often had a lower level of education or a diagnosis of a psychiatric disorder, both of which are well-known risk factors connected to ADHD in an offspring [31] and attributable to a short duration of breastfeeding [32]. We were able to include information on these factors in this report.

Lastly, the results of this prospective study further confirm the role of previously identified risk factors for ADHD such as male gender, young maternal age and low maternal education, all of which are associated with known disruptions in early neurodevelopment [31, 33]. In addition, an unwanted pregnancy was correlated with hyperactivity symptoms in the offspring at 8 years of age, but not with ADHD symptoms at 16 years. Familial factors may explain the difference between the tendencies observed here, while an unwanted pregnancy can lead to unfavourable family conditions at early ages.

The limitations of this study were as follows. First, we were unable to include family history for ADHD and those infants who had not been breastfed at all. The missing breastfeeding data may have led to an under-interpretation of the effect of breastfeeding. Second, at the age of eight we could only measure hyperactivity symptoms, which may be mixed with other symptoms besides those of ADHD, although we found that ADHD hyperactivity symptoms at the age of 8 correlated with all the ADHD symptom domains at the age of 16. Thirdly, we asked only one question regarding maternal fatigue. Fatigue is not the best estimate for stress, and it should be noted that it is distinct from stress, even though it is strongly associated with it [11]. Unwanted pregnancy is also highly loaded for impulsivity and hence the genetic risk from the mother for the outcome. Furthermore, the experience of fatigue for pregnant women in the 1980’s may have been different from what is experienced in contemporary society and at the time reports of fatigue may have been underestimated. Lastly, more than 10% of variables related to unwanted pregnancy, fatigue, and maternal education were missing and this may be a result of some questionnaires being lost. The loss is likely coincidental and the effect on the results is small.

Similarly, this study has a number of strong points. NFBC1986 is one of the largest known birth cohorts with high genetic and ethnic homogeneity. A prospective population-based cohort also means that the results are widely generalizable and recall bias is improbable. NFBC1986 enabled us to use reliable child health clinic cards for extracting information on breastfeeding. In addition, a further strength was that we were able to consider the ADHD symptom domains, which enabled us to determine whether inattentive-type ADHD and hyperactive-impulsive ADHD accounted for different results. This is a major strength, since only a few previous studies have been able to consider these symptom domains. Furthermore, we included information on parental psychiatric psychopathologies and unwanted pregnancies. To our knowledge, this is the first cohort study to include information on unwanted pregnancies in relation to offspring ADHD.

In conclusion, our findings indicate that environmental factors such as prenatal maternal stress and breastfeeding influence the pathophysiology of ADHD, although genetic factors also play a huge role in its development. Still larger studies will be needed in future to replicate and extend the present findings.

## Electronic supplementary material

Below is the link to the electronic supplementary material.

Supplementary Material 1

Supplementary Material 2

Supplementary Material 3

## Data Availability

NFBC data are available from the University of Oulu, Infrastructure for Population Studies. Please, contact the NFBC project centre (NFBCprojectcenter@oulu.fi) and visit the cohort website (www.oulu.fi/nfbc) for more information. Permission to use the data for research purposes can be applied for via the electronic material request portal. In our use of data we follow the Finnish Data Protection Act and the EU general data protection regulation (679/2016).
