# Supplementary material for: Prenatal maternal stress, breastfeeding and offspring ADHD symptoms
Source: Eur Child Adolesc Psychiatry. 2024 Apr 30;33(11):4003–11. doi: 10.1007/s00787-024-02451-5 (PMC11588867; doi:10.1007/s00787-024-02451-5)
Supplement: Supplementary file 1 — Supplementary Material 1 [file 787_2024_2451_MOESM1_ESM.docx]

Supplementary table 1. Association between maternal age during labour, fatigue, and unwanted pregnancy.

| Maternal age during labour | | | | | χ2 |
| --- | --- | --- | --- | --- | --- |
|  |  | <20 | 20-35 | >35 | p-value |
| n |  | 239 | 5783 | 751 |  |
| fatigue (%) | no | 147 (79.9) | 3764 (78.8) | 431 (70.2) | <0.001 |
|  | yes | 37 (20.1) | 1011 (21.2) | 183 (29.8) |  |
| n |  | 239 | 5783 | 751 |  |
| unwanted preg. (%) | no | 142 (70.6) | 4743 (93.5) | 595 (93.3) | <0.001 |
|  | yes | 59 (29.4) | 330 (6.5) | 43 (6.7) |  |
