# Supplementary material for: Prenatal maternal stress, breastfeeding and offspring ADHD symptoms
Source: Eur Child Adolesc Psychiatry. 2024 Apr 30;33(11):4003–11. doi: 10.1007/s00787-024-02451-5 (PMC11588867; doi:10.1007/s00787-024-02451-5)
Supplement: Supplementary file 2 — Supplementary Material 2 [file 787_2024_2451_MOESM2_ESM.docx]

|  | | |
| --- | --- | --- |
|  | Positive ADHD symptoms only at age 8 | Positive ADHD symptoms only at age 16 |
| Crude models | OR (95% CI.) | OR (95% CI.) |
| Unwanted pregnancy | 1.21 (0.62-2.51) | 0.58 (0.28-1.22) |
| Fatigue | 1.24 (0.70-2.30) | 1.68 (0.97-3.04) |
| Adjusted models |  |  |
| Unwanted pregnancy | 1.84 (0.79-4.80) | 0.83 (0.35-2.14) |
| Fatigue | 1.27 (0.67-2.49) | 1.91 (1.04-3.70)* |
| Gender (female ref.) | 0.96 (0.49-1.80) | 0.36 (0.19-0.63)* |
| Only primary education | 1.03 (0.59-1.84) | 1.32 (0.76-2.35) |
| Married | 2.14 (0.62-7.09) | 1.78 (0.53-5.59) |
| Mother´s age |  |  |
| <20 | 1.36 (0.45-3.78) | 1.84 (0.63-4.98) |
| >35 | 1.44 (0.41-4.99) | 1.18 (0.34-4.06) |

Supplementary table 2. Association between positive ADHD symptoms at both 8- and 16-year-olds to positive ADHD symptoms only at the age of 8 or 16.

| * p-value<0.05 |  |  |  |
| --- | --- | --- | --- |

Adjusted for the desirability of the pregnancy, maternal prenatal fatigue, gender of the offspring, mother´s education level, mother´s age at the time of labour, parents’ marital status, and maternal psychiatric disorders. OR = odds ratio, CI = confidence interval.
