# Supplementary material for: Prenatal maternal stress, breastfeeding and offspring ADHD symptoms
Source: Eur Child Adolesc Psychiatry. 2024 Apr 30;33(11):4003–11. doi: 10.1007/s00787-024-02451-5 (PMC11588867; doi:10.1007/s00787-024-02451-5)
Supplement: Supplementary file 3 — Supplementary Material 3 [file 787_2024_2451_MOESM3_ESM.docx]

|  |  |  |
| --- | --- | --- |
| Exclusive breastfeeding | Any ADHD symptoms at age 16 | Hyperactive symptoms at age 8 |
| Crude models | OR (95% CI.) | OR (95% CI.) |
| Breastfeeding ≥ 3 months | 1.00 (0.66-1.55) | 0.65 (0.46-0.92)* |
| Adjusted models | |  |
| Breastfeeding ≥ 3 months | 1.32 (0.78-2.37) | 0.62 (0.41-0.95)* |
| Gender (female ref.) | 1.81 (1.19-2.78)* | 3.56 (2.33-5.59)** |
| Unwanted pregnancy | 1.00 (0.38-2.19) | 1.83 (0.94-3.31) |
| Fatigue | 1.35 (0.82-2.16) | 0.97 (0.58-1.55) |
| No mother´s HS diploma | 1.69 (1.05-2.66)* | 0.76 (0.47-1.21) |
|  |  |  |
| Non-exclusive breastfeeding | |  |
| Crude models | OR (95% CI.) | OR (95% CI.) |
| Breastfeeding ≥ 6 months | 0.68 (0.48-0.95)* | 0.76 (0.54-1.06) |
| Adjusted models | |  |
| Breastfeeding ≥ 6 months | 0.65 (0.42-1.01) | 0.78 (0.51-1.19) |
| Gender (female ref.) | 1.77 (1.15-2.79)* | 3.76 (2.35-6.25)** |
| Unwanted pregnancy | 1.32 (0.53-2.83) | 2.53 (1.33-4.54)* |
| Fatigue | 1.22 (0.73-1.99) | 0.60 (0.33-1.04) |
| No mother`s HS diploma | 0.95 (0.55-1.58) | 1.09 (0.66-1.75) |
| * p-value<0.05 **p -value<0.001 | |  |

Supplementary table 3. Combined effect of prenatal maternal fatigue, unwanted pregnancy and breastfeeding duration on hyperactivity symptoms at the age of 8 and ADHD symptoms at the age of 16.

Adjusted for gender of the offspring, mother´s education level, and mother’s age at the time of labour. OR = odds ratio, CI = confidence interval.
